# Effects of post‐fire on seed germination and seedling recruitment of a generalist savanna woody species

**DOI:** 10.1111/plb.70197

**Published:** 2026-03-05

**Authors:** M. A. De Macedo, D. R. Rossatto

**Affiliations:** ^1^ Department of Biodiversity Institute of Biosciences, Sao Paulo State University (Unesp) Rio Claro Sao Paulo Brazil; ^2^ Department of Biology, Faculdade de Ciências Agrárias e Veterinárias Sao Paulo State University (Unesp) Jaboticabal Sao Paulo Brazil

**Keywords:** Field experiment, fire ecology, growth, microsites, morphological traits, seedling development

## Abstract

- Temperature and humidity are key factors influencing seed germination, varying across vegetation types and in response to disturbances such as fire. This study investigated germination, recruitment, and seedling growth of the generalist tree *Vochysia tucanorum* Mart. (Vochysiaceae) in natural environments of southern Brazil, including savanna, savanna – forest transition, and forest vegetation.
- Three months after a fire event, we established 12 nurseries for treatment (four per vegetation type, each containing 15 seeds) and monitored environmental conditions and seedling development from December 2022 to July 2023. Data on temperature, humidity, germination, mortality, seedling height, and cotyledon thickness were compared between burn and unburn treatments.
- Average temperature differed among vegetation types but not between burned and unburned areas, while humidity increased by 28% in burned plots. The number of seeds that germinated was significantly higher in burned areas across all vegetation types. Seedlings in the savanna were smaller (mean 16 mm) than those in the transition (29.6 mm) and forest sites. Cotyledon thickness varied over time and between treatments but was not affected by vegetation type.
- Despite the wide ecological distribution of *V. tucanorum*, germination and recruitment were rare events. The limited germination in savanna vegetation indicates that microclimatic constraints, particularly reduced water availability, restrict seedling establishment. Once germinated, seedling growth depends largely on cotyledon reserves, which deplete more slowly in burned environments. These results improve our understanding of post‐fire regeneration in a generalist wood species and demonstrate how fire and microclimatic conditions jointly influence recruitment dynamics in fire‐prone environments.

## Graphical abstract

Vegetation type and microclimate shape recruitment of a generalist tree seedling, while fire history alters cotyledon use over time.

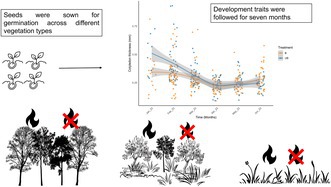

## INTRODUCTION

The impact of fire on vegetation is influenced by various factors (Hoffmann 2000), including plant functional traits (Chiminazzo *et al*. 2023), plant height and life stage and vegetation structure (Midgley 1996). During the seed stage, a population's ability to expand into new areas relies on several critical processes. First, successful seed dispersal to suitable microsites is a prerequisite for germination (Schupp 1995; Schupp *et al*. 2010). After dispersal, germination depends on favourable microclimatic conditions, particularly light availability associated with canopy openings and adequate soil moisture (Souza *et al*. 2015). Suitable microsites often are a combination of resource availability, environmental conditions, and competition levels (Schupp 1995). Among Tropical Brazilian Atlantic Forest species, around 67.7% of seeds require light for germination, while 21.5% can germinate in both light and shade, which may enable, in the case of generalist species, to establish in a series of environments, including savanna environments (Souza *et al*. 2015).

Fire events significantly alter vegetation structure and microclimatic conditions (Marcolin *et al*. 2019), impacting factors like temperature, humidity, and light availability (Ma *et al*. 2010). In unburned savannas, seeds landing on dense ground‐layer often face challenges such as reaching the soil, which can reduce their chances of successful germination (Coutinho 1977). However, the consumption of ground layer vegetation by fire can increase seed recruitment by allowing seeds to reach the soil more easily (Coutinho 1977). Although fire‐induced removal of surface cover can temporarily increase light and soil nutrient availability (Blair 1997; Setterfield 2002), the resulting burned environment may also create conditions that are unfavourable for seedling establishment. For instance, in burned savanna areas, low establishment rates are commonly observed during the first year for tree seedling growth (Hoffmann 2000; Scott *et al*. 2010). Such reduced recruitment can arise from multiple factors, including limited seed supply (Dairel & Fidelis 2024), competition with vigorous vegetative regrowth, and microclimatic changes such as increased temperature and reduced moisture (Newberry *et al*. 2020).

Meanwhile, transition zones between forest and savanna typically exhibit a less dense ground‐layer, with abundant shrubs, and higher tree density compared with open savannas (Abreu *et al*. 2017; Newberry *et al*. 2020). Forests, in contrast, are characterized by closed canopies, low abundance of graminoids and herbaceous species, and thick litter layer. These structural differences lead to markedly distinct fire responses among vegetation types (Newberry *et al*. 2020). Consequently, the effects of fire vary across savannas, transition zones, and forests, relying on species composition and microclimatic conditions (Hoffmann 1998; Hoffmann *et al*. 2004; Newberry *et al*. 2020). To understand how a tree species, which acts as a nucleator, germinates across different vegetation types and how seedling recruitment is affected, it is crucial to consider microsites and their role in supporting the establishment of new individuals from dispersed seeds (Hoffmann *et al*. 2004). Despite the importance of field germination studies in post‐fire conditions, such research is limited. Studying recruitment patterns of a generalist tree in both open savannas and closed forests can shed light on the restrictions and opportunities for post‐fire recruitment and the role of vegetation density.

Beyond germination, evaluating early development and establishment of seedlings is essential for understanding its recruitment in different vegetations. Seedlings depends on vital structures, such as cotyledons and true leaves, to enhance their survival chances until full establishment (Kitajima 1994; Garwood 1996). Successful germination and rooting help ensure that young plants avoid sun exposure and establish themselves in the soil (Silva & Castro 1989). Understanding these dynamics in fire‐prone ecosystems like savannas, which feature adaptive traits to withstand fire, can reveal how environmental filters shape vegetation (Bond & Keeley 2005; Pausas 2017). Traits such as heat tolerance (Ribeiro *et al*. 2013; Zirondi *et al*. 2019; Macedo *et al*. 2021), dormancy break by high temperatures, and germination stimulated by smoke or ash (Daibes *et al*. 2018; Motta *et al*. 2024) are well documented for savanna species. Although these traits highlight strategies that plants use to cope with fire‐prone environments, understanding post‐fire dynamics also requires assessing seedling performance in situ. Evaluating seedling performance across unburned and burned areas in different vegetations – savanna, forest–savanna transition, and forest – can be achieved through detailed measurements of morphological traits, such as cotyledon size, thickness, height, and leaf number (Garwood 1996). Such measurements allow for a deeper understanding of the long‐term effects of fire on the recruitment of tree species, their post‐fire survival strategies, and the dynamics of seedling establishment in fire‐affected and unaffected areas.

We expect forests to show higher germination and recruitment of seeds than savannas because their have a more buffered understory microclimate – driven by higher canopy closure – which helps stabilize temperature and moisture, favouring early establishment. Thus, this study aims to evaluate germination, seedling growth and survival of a model species: *Vochysia tucanorum* Mart. (Vochysiaceae) after a post‐fire event. Specifically, we assessed seed germination across three vegetation types—savanna, forest–savanna transition, and forest – over 6 months to determine germination rates, recruitment success, and seedling development. We addressed the following research questions:

1. Does vegetation type and treatment (burned *versus* unburned) influence the germination of *Vochysia tucanorum* seeds?
2. Does temperature and humidity affect germination and subsequent development of the species?
3. Is the use of cotyledon resources influenced by month and vegetation type?

## METHODS

### Study site

The study was conducted at Santa Bárbara Ecological Station (SBES), located at 22° 46′ 30″ ‐ 22° 50′ 30″ S and 49° 10′ 30″ ‐ 49° 15′ 30″ W, southern Brazil, São Paulo state, Brazil. The reserve is characterized by a hot and humid climate (Cwa), with an annual rainfall of approximately 1424 mm and an average annual temperature of 20.9 °C. The SBES features a variety of vegetation types, including savanna, semi‐deciduous seasonal forests, and forest‐savanna ecotones. In savannas, grass makes up less than 10% of cover places that have not burned, but rises to about 80% in burned areas. Common plant families include Poaceae, Fabaceae, and Verbenaceae (Abreu *et al*. 2017). Additionally, other plants like forbs and subshrubs cover around 40% of unburned areas and 50–60% of burned areas (Pilon *et al*. 2018). At SBES, savanna–forest transition vegetation features a tree basal area of 11.94 m^2^/ha and grass biomass of less than 200 g/m^2^. Significant plant families found in these areas include Asteraceae, Poaceae, Verbenaceae, Fabaceae, and Lauraceae. On the other hand, forest areas have closed canopies and lack graminoid layer, shows tree cover exceeding 68%. Common families in these forests are Myrtaceae, Asteraceae, Fabaceae, and Bignoniaceae. The area selected for the experiment had a zero‐fire policy, although there was an accidental fire in 2011 (Pilon *et al*. 2018). In 2014, for research purposes, areas of forest, forest–savanna transition, and savanna were delineated with an annual fire regime in the dry season aimed at maintaining open systems. Each of the vegetation types mentioned above has characteristics that can either facilitate or hinder germination, development, and recruitment. For generalist woody species, many of these characteristics can facilitate the germination and recruitment of this vegetation.

### Model species

*Vochysia tucanorum* Mart. ‐ Vochysiaceae is a widely and generalist distributed tree along the Cerrado domain (Ratter *et al*. 2003). It is an evergreen species, with anemocoric dispersion, that can be found in both forest and savanna formations, and it has fruiting and seed dispersal from August to September (Lorenzi 1992). This species has a singular historical factor that makes it easier for this species to be present in both forest and savanna environments, because the *Vochysiaceae* group is older (Gonçalves *et al*. 2020) than the emergence of the savanna vegetation of South America (Ledru 2002). Because of that, this taxonomic group is abundant in the experimental areas and has a high ecological value as a nucleating species to promote the recruitment of other tree species (Abreu *et al*. 2021).

### Experimental trials

Three months after fire (November to June), in each vegetation type (savanna, forest–savanna transition, and forest), a set of four nurseries with 15 seeds were installed to monitor germinative and post‐germinal processes during the data collection period. This period was chosen because the species had already completed its seed dispersal period (Lorenzi 1992), ensuring that no additional seeds would be introduced after the installation of the nurseries. In this study, a set of four nurseries was considered the sampling unit because of the high variability of germination and survival of the seeded individuals. To protect the seeds from being carried away by ants and/or rainwater, transparent plastic protections measuring 10 cm high, 20 cm long, and 15 cm wide were installed. Throughout the experimental period (December 2022 to July 2023), soil surface temperature and humidity were monitored using a *HOBO U23‐00x* sensor equipped with a ceramic protective shield. The sensor was placed directly on the soil surface to measure temperature and humidity experienced by seeds during germination and early development. This setup ensured exposure to direct solar radiation, replicating natural field conditions. These parameters were recorded every hour in each vegetation type (savanna, forest–savanna transition, and forest) for each treatment. Germination was counted after observing radicle protrusion or epigeal germination. In the first month after seeding (November), germination was monitored every 15 days, and thereafter, it was monitored once per month. Seed mortality was evaluated and considered when the seed appeared with the endosperm and embryo consumed or without germination at the end of the period of observation. In seedlings, mortality was considered when the aerial part was lost completely.

To evaluate the investment in seedling establishment after germination, we measured the height once the cotyledons opened. This helped avoid errors caused by uneven soil and variable hypocotyl growth. We measured the height based on the highest point of the apical meristem of the cotyledons if there were no leaves. For cotyledon thickness, which indicates resource storage (Kitajima 1992), we used a digital calliper to measure at the midpoint of the cotyledon. We also counted the leaves, if they were present, to assess the investment in leaf growth.

### Statistical analysis

Differences in temperature and humidity among vegetation types and fire treatments were analysed using generalized additive models (GAMs) with a Gaussian distribution (*family = gaussian*) implemented in *mgcv* (Wood 2003). Each observation corresponded to the mean value per month, vegetation type, and treatment. Models included vegetation type and treatment as fixed effects, without random or smooth terms, to test differences in mean environmental conditions between burned and unburned plots.

Germination and mortality counts were modelled with negative binomial generalized linear models (GLMs) using a log link (*MASS* package; Venables & Ripley 2002). Predictors included treatment, vegetation type, and month, with a Vegetation × Month interaction to test temporal variability. Predicted germination and mortality probabilities were obtained using the predict() function (*type = ‘response’*). Model fit and dispersion were assessed following Zuur *et al*. (2009).

Continuous traits (height and cotyledon thickness) were analysed using generalized additive mixed models (GAMMs) with Gaussian distribution and identity link (*mgcv*). Fixed effects included treatment, vegetation type, and when relevant, month. A random‐effect smooth for parcel (*s(Parcel, bs = ‘re’)*) accounted for repeated measures and spatial grouping. Model parameters were estimated by REML, and adequacy was evaluated via residual diagnostics and explained deviance (R^2^).

Additional continuous traits (*e.g*., leaf number) were analysed with linear mixed‐effects models (LMMs; *lme4*), including treatment, vegetation, month, and their interactions as fixed effects, and individual identity as a random intercept. Residuals were inspected for normality and homoscedasticity. Post hoc contrasts were performed using *emmeans* (Lenth 2024). The numerical values are presented as mean ± standard error (SE).

## RESULTS

### Environmental conditions

**Fig. 1:**
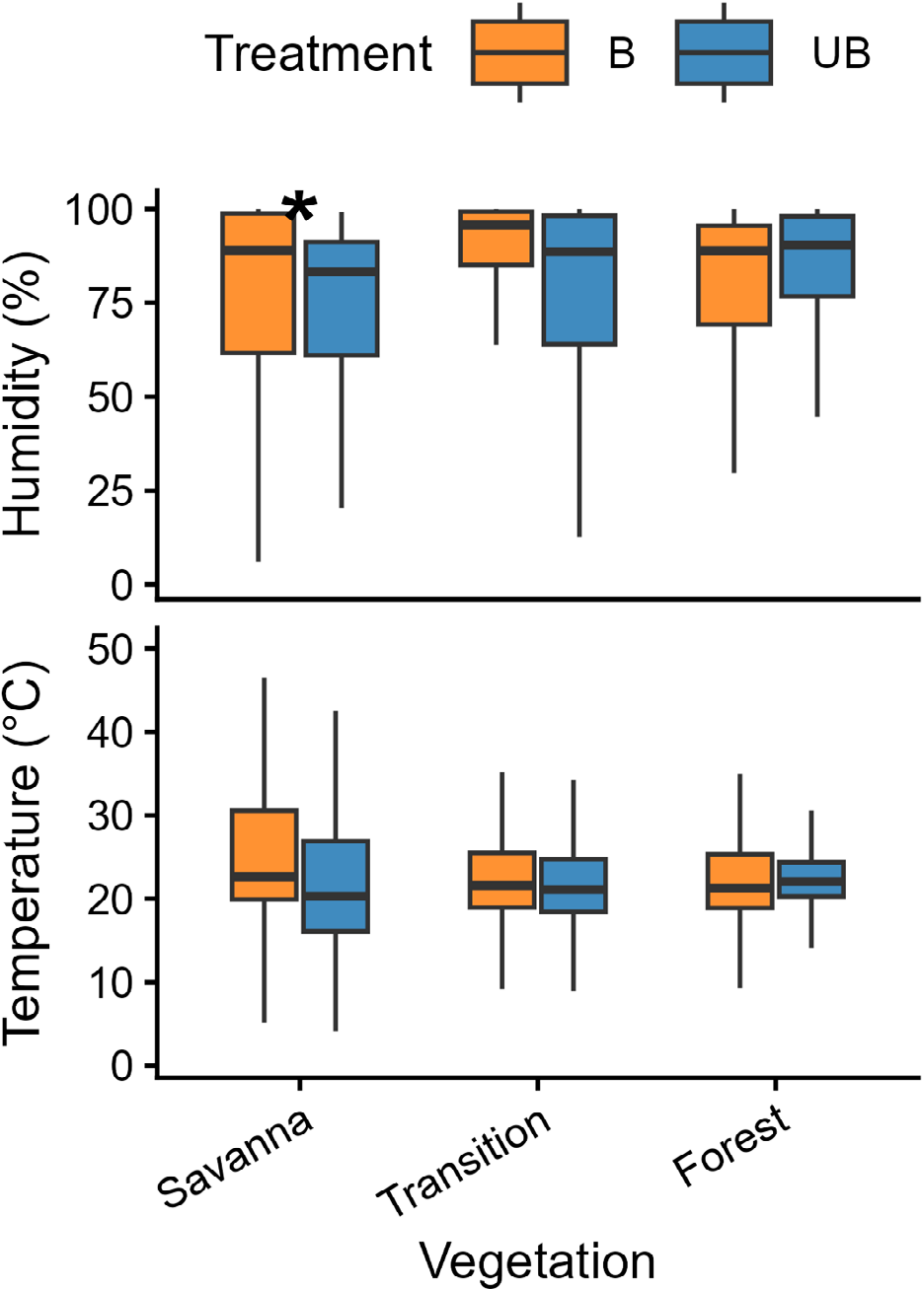
Variation in relative humidity (%) and temperature (°C) across vegetation types under burned (B) and unburned (UB) treatments. Boxplots show observed values, with medians indicated by central lines. Differences among conditions were tested using Gaussian GAMs; asterisks (*) indicate significant effects (*P* < 0.05).

We found significant differences in mean temperature among vegetation types when considering treatment and month, though no differences were observed between treatments. Unburned savanna areas recorded the highest mean temperature (26.67 ± 13.69 °C), followed by burned savanna plots (25.40 ± 9.24 °C) and unburned forest (22.06 ± 3.61 °C). The forest–savanna transition showed the lowest mean temperatures, with unburned plots averaging 21.46 ± 6.29 °C and burned plots 21.99 ± 5.27 °C (Table 1 and see Fig. 1 for the distribution of observed values).

**Table 1:** Results of generalized additive model (GAM) for temperature and humidity in different vegetations.

Vegetation	Estimate	t‐value	*P*‐value
Savana	28.65	4.22	<0.0001
Transition	−10.79	−2.75	<0.0001
Forest	−11.39	−2.91	<0.0001
Unburned	0.67	0.77	0.44

For each vegetation type, use savanna burning as a parameter to compare. The model applied temperature as a function of vegetation and treatment; the model explained 89% of the variables (R = 89%) without a smooth effect.

Humidity differed between treatments and vegetation types. The highest mean value (88%) was found in the burned forest–savanna transition vegetation (Table 2; *P* = 0.04), followed by forest (68.7%) and unburned savanna (23.79%). In general, humidity increased by 28% in burned areas (*P* < 0.001; see Fig. 1 for the distribution of observed values).

### Germination

**Fig. 2:**
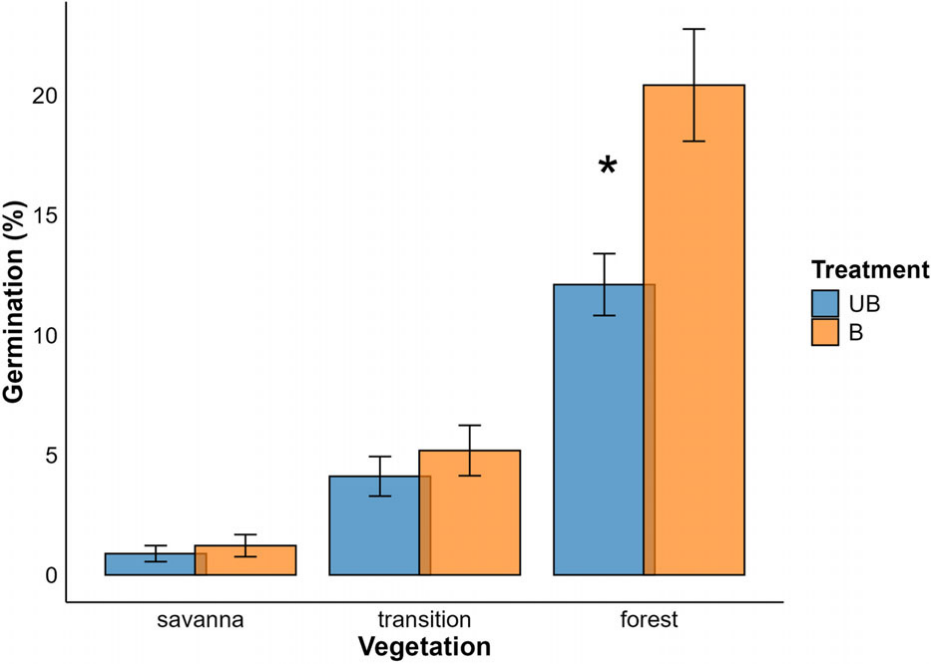
Mean germination (%) of seeds in burned (B, orange) and unburned (UB, blue) plots across vegetation types. Data were analysed using GLMs with negative binomial distribution. Germination was higher in burned plots (*P* < 0.05). Burned areas were affected by fire in August 2022, and seeds were sown 3 months later. The symbol * showed the significant effect *P* < 0.05 between treatments.

**Fig. 3:**
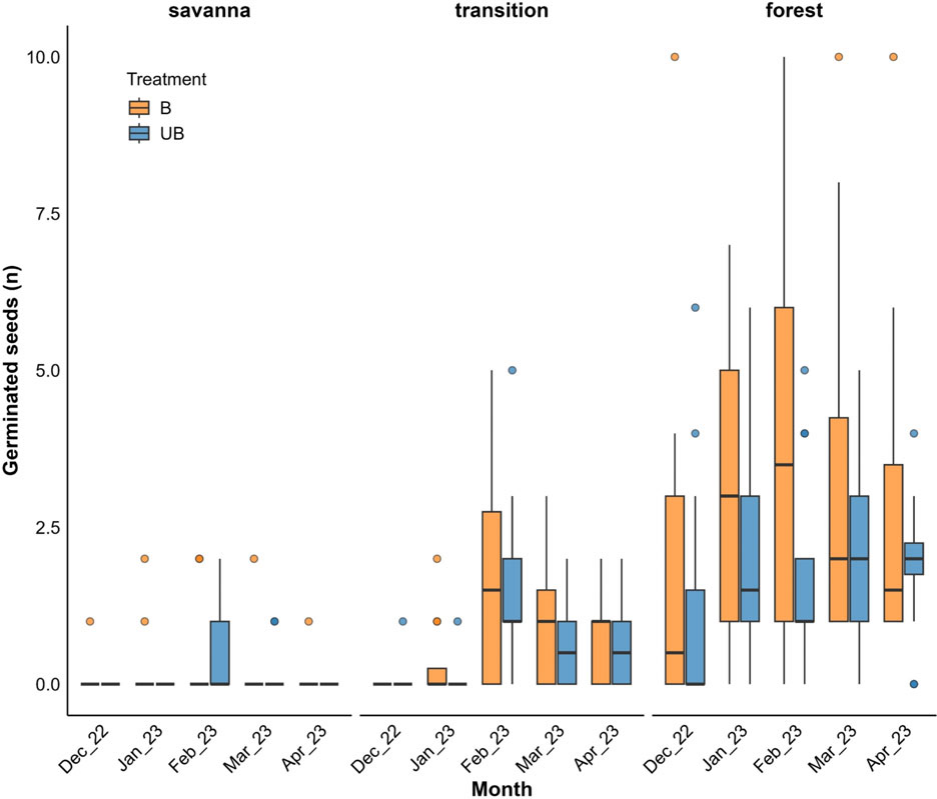
Monthly number of germinated seeds (n) in burned (B, orange) and unburned (UB, blue) plots across vegetation types. Boxplots represent observed field data. Seeds were sown in November, with 15 seeds per nursery, four nurseries per replicate, and three replicates per treatment and vegetation type (total = 180 seeds). Effects of treatment, vegetation type, month, and their interaction were tested using GLMs with a negative binomial distribution. The figure illustrates temporal variation and differences among vegetation types and treatments. Statistical significance refers to the time × vegetation × treatment interaction (*P* < 0.05).

For germination, vegetation types had significant effects (Table 2). Seeds were sown in November, with a total of 180 seeds per treatment and vegetation type, it tended to be higher in burned plots; however, this increase was statistically significant only in the forest vegetation (Fig. 2; *P* < 0.005). Time also had a significant effect, interacting with both vegetation and treatment (Fig. 3; *P* < 0.005).

**Table 2:** Results of the generalized linear model (GLM) with a negative binomial distribution evaluating average seed germination (%) as a function of vegetation type, fire treatment, temperature, and humidity.

Variables	Estimate	t‐value	*P*‐value
Temperature	−0.14	0.23	0.53
Humidity	−0.09	0.05	0.04

The model explained 75% of the variance (R^2^ = 0.75). Only humidity showed a significant effect (*P* < 0.05).

Across all vegetation types, the highest observed germination values occurred in February. In savanna, the maximum germination occurred in unburned plots, reaching 3.33% during that month (Fig. 3). In the savanna–forest transition, the highest germination rate was observed in burned plots, reaching 12.2% (*P* < 0.01). In forest vegetation, mean germination across the study period reached a maximum of 26.1% (*P* < 0.01), also in burned plots (Fig. 2). Model predictions indicated that in the savanna, germination probability ranged from 0.15 (≈15%) in burned plots to 0.08 (≈8%) in unburned plots. In the savanna–forest transition, predicted probabilities were 0.53 (≈53%) and 0.46 (≈46%) for burned and unburned plots, respectively (Table S1). For the forest, germination probability increased from 0.64 (≈64%) to 0.75 (≈75%) after burning (*P* < 0.001; Fig. 2; Table S1). Overall, these results indicated enhanced germination performance in burned plots, particularly in forest vegetation, where the difference between treatments was most pronounced.

### Seedling growth

Seedling height was modelled using a Gaussian GAMM that included the interaction between Treatment and Vegetation, as well as Month, as fixed effects. A random smooth term for Parcel (s(Parcel, bs = ‘re’)) was added to account for temporal dependence among plots. The model explained 44.4% of the deviance (adjusted R^2^ = 0.43; n = 694). Vegetation type and sampling month had strong effects on seedling height (*P* < 0.001), while treatment alone was not significant (*P* = 0.99). The interaction between treatment and vegetation was also non‐significant (*P* > 0.1), although seedlings were consistently taller in forest and transition plots than in the savanna. The random effect of Parcel was highly significant (*P* < 0.001), indicating substantial spatial variability in seedling growth among plots.

**Fig. 4:**
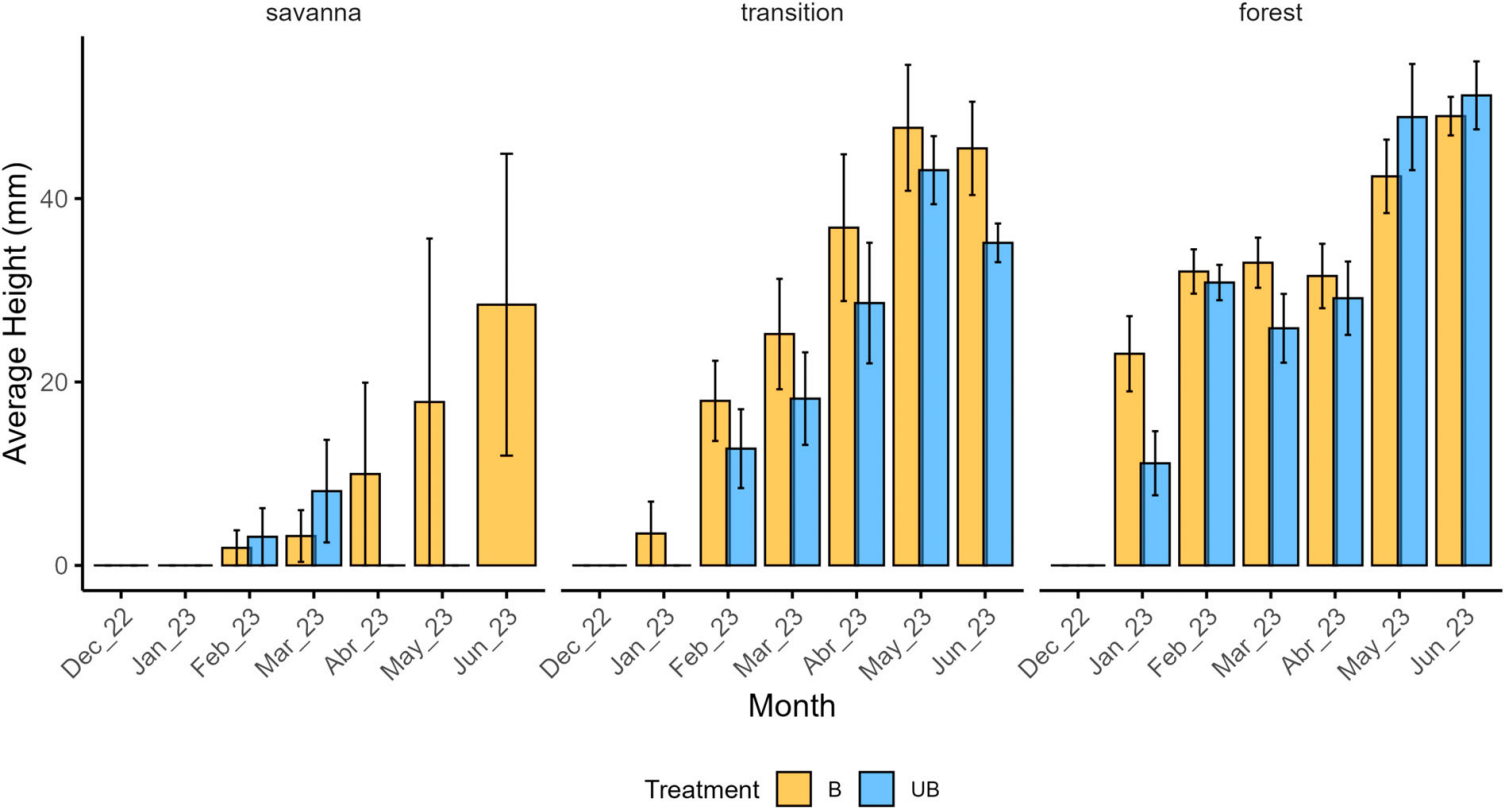
Average height (mm) of *Vochysia tucanorum* seedlings over 7 months in burned (B, orange) and unburned (UB, blue) plots. Analyses used generalized additive mixed models (GAMMs) with a Gaussian distribution, including treatment, vegetation type, and month. Height increased over time (*P* < 0.001) and was higher in transition and forest than in savanna.

In the savanna, seedling height could not be reliably compared between treatments because mortality was extremely high, leaving only a single surviving individual in the burned plots to measure the growth. As a result, statistical differences in height could not be evaluated for this vegetation type. In contrast, in the forest–savanna transition, seedlings in burned plots were substantially taller (mean = 50.4 mm) than those in unburned plots (mean = 35.7 mm; *P* = 0.013). In forest vegetation, height differences between treatments were not significant (*P* = 0.062). Across vegetation types, seedling height differed significantly (*P* < 0.001): seedlings from the savanna were shorter (mean = 16 mm) than those from the transition zone (mean = 50.4 mm) and forest (mean = 29.6 mm; Fig. 4).

### Cotyledon traits

The opening of cotyledons occurred from January to July 2023. During this period, seedlings grew an average of 32 mm. The highest growth rate was observed 3 months after seeding, with approximately 11.4 mm. After this growth peak, the rate remained below 5 mm.

**Fig. 5:**
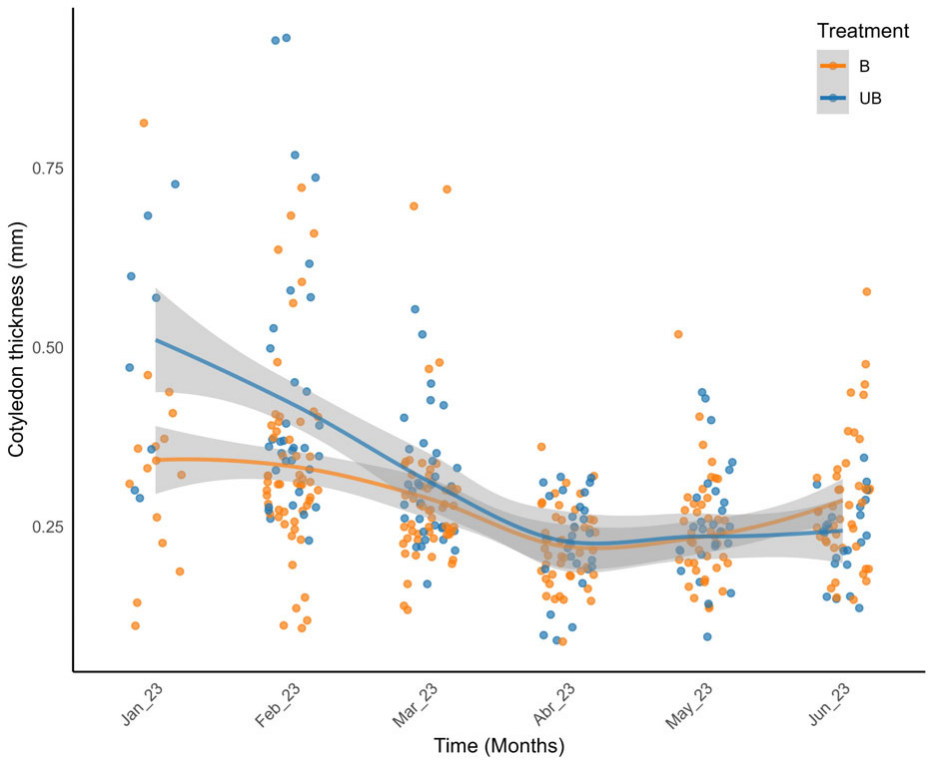
Cotyledon thickness (mm) of *Vochysia tucanorum* seedlings under burned (B, orange) and unburned (UB, blue) treatments over time. GLMs with Gaussian distribution showed temporal variation across vegetation types, with thicker cotyledons in transition and forest plots during February and May (*P* < 0.05).

Development traits, such as cotyledon thickness (mm/month), responded significantly to treatment and time, but not to vegetation type. Although vegetation‐specific values are shown in Table 3 for descriptive purposes, statistical significance was detected only when temporal variation was incorporated into the model. We found a decrease in the thickness of cotyledons between treatments with an interaction of time. Over time, cotyledon thickness decreased in both treatments (Fig. 5). Unburned areas exhibited the highest observed thickness values in January (≈0.50 mm), followed by a marked decline over subsequent months. The greatest loss in unburned plots occurred between February and March (≈0.11 mm; *P* < 0.001), whereas in burned plots the highest loss occurred between March and April (*P* < 0.001). Between March and April, cotyledon thickness in unburned areas remained relatively stable, whereas burned areas showed a temporary increase in thickness between May and June (≈0.04 mm; Fig. 5). In each vegetation, development occurs in different ways. Treatments interfered with seedling development, even though there was no significant difference when compared overall. When we evaluated each development trait, it was possible to see differences in growth strategies. In unburned savanna areas, the correlation between time (months) and height growth, leaf development, and cotyledon thickness was not significantly associated. However, the taller the seedling was, the greater the chance of having leaves (R = 0.84; t = 11.21; *P* < 0.001; Table S2) and increased cotyledon thickness (R = 0.97; t = 30.93; *P* < 0.001).

**Table 3:** Average cotyledon thickness (mm) of *V. tucanorum* seedlings sown in vegetation that was burned in August 2023 and in unburned vegetation.

Vegetation	Unburned (mm)	Burned (mm)
Savanna	0.27 ± 0.02	0.30 ± 0.08
Transition	0.29 ± 0.07	0.27 ± 0.08
Forest	0.31 ± 0.15	0.28 ± 0.11

The seeds were deposited in November and only began to germinate in December.

Nevertheless, in burned areas, time (months) was positively associated with height growth (R = 0.48; t = 4.22; *P* < 0.001), cotyledon thickness (R = 0.38; t = 3.15; *P* < 0.002), and an increase in the number of leaves (R = 0.39; t = 3.26; *P* < 0.001). For both treatments, the increase in cotyledon thickness was significantly and positively correlated with the number of leaves (Table S2) and seedling height. Unlike savanna vegetation, seedlings in the savanna–forest transition and forest vegetation did not show differences in these development traits. The number of leaves increased over time and varied across vegetation. The savanna had fewer leaves compared with the savanna–forest transition and forest (Table S2).

## DISCUSSION

We hypothesize that once *Vochysia tucanorum* starts to germinate, its development is influenced by vegetation type in which it grows. As a generalist species, it shows notable differences in recruitment potential between closed and open areas. The number of germinated seeds was generally higher in burned areas compared with unburned ones (Fig. 2), except in the savanna, where they were similar. This can be associated with vegetation type, which exhibits different burn efficiencies. In forest vegetation, burn efficiency is typically low due to higher humidity levels and a lack of fine dry fuel (Newberry *et al*. 2020). This makes it difficult for fire to spread widely, leading to patchy burning. However, as burn frequency increases, forested areas become more prone to developing gaps and retaining smoke for longer periods compared to open areas (Certini 2005). In such burned environments, fire by‐products such as ash can enhance germination (Zuloaga‐Aguilar *et al*. 2011). In the field, we observed that burned forest areas exhibited the highest germination rates for this species, which cannot be directly attributed to microclimatic variables, as humidity and temperature did not show significant differences between treatments. The increase in germination is likely associated with factors like the diffusion of smoke into the soil (Ghebrehiwot *et al*. 2011). Studies suggest that ash and smoke can enhance germination, with their effects lingering in the soil for months (Coutinho *et al*. 1982; Zuloaga‐Aguilar *et al*. 2011). While the interaction between post‐fire products and microclimatic factors remains understudied, our findings indicate their potential as selective factors. Furthermore, in burned areas, clearings are opened, facilitating the competitive process of these seedlings.

Germination and recruitment of *V. tucanorum* seedlings may also be influenced by soil characteristics. The cumulative data on germination and mortality showed varying benefits of different vegetation types for germination over time, with mortality rates increasing as time progressed. Mortality can occur at both the seed and seedling stages. In the savanna environment germination rate was very low (3.33%), with many seeds failing to germinate. One key challenge is the ability to germinate in an environment with high‐temperature variability and intense sun exposure, which can desiccate seeds, hindering germination (Ribeiro & Borghetti 2014). Temperatures in savanna ranged from12.3 °C to 42 °C. Even native species to this environment rarely achieve successful germination due to the risk of drying out (Sales *et al*. 2013; Ribeiro & Borghetti 2014). Soil properties also play a role in germination, as smaller seeds (<0.5 mg) may face difficulties in retaining sufficient moisture, especially in sandy soils, leading to water stress (Kos & Poschlod 2008). This water stress is most pronounced in savanna and diminishes in forest–savanna transition and forest areas. The increased humidity in these more closed systems improves seedling survival, as survival rates rise with greater wood and ground cover (McLaren & McDonald 2003).

According to Barbosa *et al*. (1999), the seeds of *V. tucanorum* are not photoblastic, meaning they can germinate in both light and dark conditions. Their study found that the highest germination percentage occurred at 30 °C under light conditions. Our field results, however, suggest that humidity plays a more critical role than temperature in field germination. This species can germinate across different environments, but its success in savannas is limited (Germination = 3.33%), occurring only under extreme conditions such as low humidity (12%) and high temperatures (>40 °C). The spatial distribution of adult *V. tucanorum* is more influenced by water availability and seed dispersal ability than by light (da Costa & dos Santos 2016), which aligns with our statistical analysis showing that humidity restricts germination more than temperature (Table 1).

Survival in savannas interplay of genetic adaptations and historical biogeographic factors linked to population origin (Fortunel *et al*. 2016). While generalist species exhibit phenotypic plasticity that enables persistence across both savanna and forest habitats, their success remains constrained by microclimatic thresholds – particularly temperature and humidity extremes (Rossatto *et al*. 2013). In forest–savanna transition zones and closed‐canopy forests, microclimatic stability buffers temperature variability: for instance, daily fluctuations are minimal, ranging from 23.2 to 24.1 °C in transition areas and 20.6–24.3 °C in forests. This thermal stability maintains consistent humidity levels, a critical determinant of seed germination and seedling establishment (Hovenden *et al*. 2008). Experimental evidence from Australian ecosystems demonstrates that even moderate temperature increases (*e.g*., >2–3 °C above ambient) can significantly reduce germination rates and early seedling survival (Hovenden *et al*. 2008). However, post‐establishment vulnerability persists; seedlings face high mortality risks during seasonal droughts due to limited root development and water retention capacity.

Development traits of cotyledons displayed differences between treatments over time, but not across vegetation types. In this case,trait differences may be associated with the water supply. Cotyledons play a crucial role in seedling establishment, providing essential carbohydrates, nutrients, and hormones for growth (Milberg & Lamont 1997). *Vochysia tucanorum* seedlings have phanerocotylar cotyledons, which emerge from the seed coat to perform photosynthesis (Kitajima 1992; Garwood 1996), and remained functional for at least 5 months in our experiment. These cotyledons support seedling survival and growth, regardless of vegetation type, but in burned areas, stored energy resources are depleted more slowly compared with unburned areas. This difference may be related to the vegetation structure: in unburned areas, space, and light are limited, whereas burned areas provide greater availability of these resources (Coutinho *et al*. 1982). This balance between stored resource consumption and photosynthetic production allows for a more efficient strategy for growth in burned environments.

Therefore, cotyledons can be considered crucial storage structures as they offer a survival advantage during dry periods or when other structures are damaged (Milberg & Lamont 1997). The benefits provided by cotyledons are enhanced by the presence of leaves, which contribute to carbon synthesis, thus increasing seedling fitness, particularly during the dry season when reserves are vital for survival. Cotyledons can persist in *V. tucanorum* seedlings for at least 4 months (Barbosa *et al*. 1999) and in some species, up to 42 weeks under shaded conditions (Augspurger 1984). Seedlings with both cotyledons and leaves, have multiple mechanisms for persistence and growth, increasing their vigour (Nelson & Larson 2015). For successful recruitment, a well‐developed root system is also essential for accessing water and nutrients (Michael & Peter 2000).

## CONCLUSIONS

The generalist tree species *Vochysia tucanorum* showed limited germination in savanna vegetation, regardless of fire treatment. Although generalist species are typically efficient at expanding across forest and forest–savanna transition gradients, germination events of *V. tucanorum* declined sharply when seeds were dispersed into savanna habitats. This indicates that despite its abundance and ecological versatility, germination is a rare event for this species under savanna conditions. The reduced germination and early seedling development appear to be related to the low water availability in this environment. Similar patterns have been observed in other vegetation types, but germination success was notably higher in the transition and forest areas. Once germination occurs, seedling growth depends largely on cotyledon nutrient reserves. In burned environments, the depletion of these reserves occurs more slowly than in unburned areas, suggesting that post‐fire conditions influence resource‐use strategies in *V. tucanorum* seedlings.

## AUTHOR CONTRIBUTIONS

MAM, data collection, screening, analysis, and writing. DRR, experimental design, writing, and correction.

## CONFLICT OF INTEREST STATEMENT

No potential conflict of interest was reported by the authors.

## Supporting information


**Table S1.** Predicted probabilities of seed germination and mortality obtained from the generalized linear model (GLM) fitted with a binomial distribution and logit link function. The model evaluated the effects of fire treatment and vegetation on germination outcomes. Predictions were generated using the predict () function with the argument type = ‘response’, which provides the estimated probability (on the response scale) of germination or death for each combination of factors based on the fitted model.

**Table S2.** Monthly median number of leaves per seedling across vegetation types and treatments (B = burned; UB = unburned) over the 7‐month study period, accounting for repeated measurements of the same individuals. *N. seedlings* indicate the number of seedlings observed per month (number total expected: 60).

## References

[plb70197-bib-0001] Abreu R.C.R. , Durigan G. , Melo A.C.G. , Pilon N.A.L. , Hoffmann W.A. (2021) Facilitation by isolated trees triggers woody encroachment and a biome shift at the savanna–forest transition. Journal of Applied Ecology, 58, 2650–2660.

[plb70197-bib-0002] Abreu R.C.R. , Hoffmann W.A. , Vasconcelos H.L. , Pilon N.A. , Rossatto D.R. , Durigan G. (2017) The biodiversity cost of carbon sequestration in tropical savanna. Science Advances, 3, e1701284. 28875172 10.1126/sciadv.1701284 PMC5576881

[plb70197-bib-0003] Augspurger C.K. (1984) Light requirements of neotropical tree seedlings: a comparative study of growth and survival. Journal of Ecology, 72, 777.

[plb70197-bib-0004] Barbosa A.R. , Yamamoto K. , Valio I.F.M. (1999) Effect of light and temperature on germination and early growth of *Vochysia tucanorum* Mart., Vochysiaceae, in cerrado and forest soil under different radiation levels. Revista Brasileira de Botânica, 22, 275–280.

[plb70197-bib-0005] Blair J.M. (1997) Fire, in availability, and plant response in grasslands: a test of the transient maxima hypothesis. Ecology, 78, 2359–2368.

[plb70197-bib-0006] Bond W.J. , Keeley J.E. (2005) Fire as a global ‘herbivore’: the ecology and evolution of flammable ecosystems. Trends in Ecology & Evolution, 20, 387–394. 16701401 10.1016/j.tree.2005.04.025

[plb70197-bib-0007] Certini G. (2005) Effects of fire on properties of forest soils: a review. Oecologia, 143, 1–10. 15688212 10.1007/s00442-004-1788-8

[plb70197-bib-0008] Chiminazzo M.A. , Bombo A.B. , Charles‐Dominique T. , Fidelis A. (2023) Bark production of generalist and specialist species across savannas and forests in the Cerrado. Annals of Botany, 131, 613–621. 36651635 10.1093/aob/mcad014 PMC10147323

[plb70197-bib-0009] Coutinho L.M. (1977) Aspectos ecológicos do fogo no cerrado. Ii ‐ as queimadas e a dispersão de sementes em algumas espécies anemocóricas do estrato herbáceo‐subarbustivo/ecological aspects of fire in the cerrado. Ii ‐ fire and seed dispersion in some anemochoric species of the herbaceous layer. Boletim de Botânica da Universidade de São Paulo, 5, 57–63.

[plb70197-bib-0010] Coutinho L.M. , De Vuono Y.S. , Lousa J.S. (1982) Aspectos ecológicos do fogo no cerrado: IV. A época da queimada e a produtividade primária líquida epigéia do estrato herbáceo subarbustivo. Revista Brasileira de Botânica, 5, 37–41.

[plb70197-bib-0011] da Costa R.C. , dos Santos F.A.M. (2016) Spatial patterns of two co‐occurring savanna and forest tree species in a dense fire‐protected savanna fragment. Acta Botânica Brasílica, 30, 577–584.

[plb70197-bib-0012] Daibes L.F. , Gorgone‐Barbosa E. , Silveira F.A.O. , Fidelis A. (2018) Gaps critical for the survival of exposed seeds during Cerrado fires. Australian Journal of Botany, 66, 116–123.

[plb70197-bib-0013] Dairel M. , Fidelis A. (2024) Fire stimulates seedling recruitment from the seed bank in the Cerrado. Journal of Vegetation Science, 35, e13278.

[plb70197-bib-0015] Fortunel C. , Paine C.E.T. , Fine P.V.A. , Mesones I. , Goret J.Y. , Burban B. , Cazal J. , Baraloto C. (2016) There's no place like home: seedling mortality contributes to the habitat specialisation of tree species across Amazonia. Ecology Letters, 19, 1256–1266. 27600657 10.1111/ele.12661

[plb70197-bib-0016] Garwood N. (1996) Functional morphology of tropical tree seedlings. In: Swaine M.D. (Ed), The ecology of tropical forest tree seedlings. Parthenon Publishing Group, London, UK, pp 59–129.

[plb70197-bib-0017] Ghebrehiwot H.M. , Kulkarni M.G. , Light M.E. , Kirkman K.P. , Van Staden J. (2011) Germination activity of smoke residues in soils following a fire. South African Journal of Botany, 77, 718–724.

[plb70197-bib-0018] Gonçalves D.J.P. , Shimizu G.H. , Ortiz E.M. , Jansen R.K. , Simpson B.B. (2020) Historical biogeography of Vochysiaceae reveals an unexpected perspective of plant evolution in the Neotropics. American Journal of Botany, 107, 1004–1020. 32643810 10.1002/ajb2.1502

[plb70197-bib-0019] Hoffmann W.A. (1998) Post‐burn reproduction of woody plants in a neotropical savanna: the relative importance of sexual and vegetative reproduction. Journal of Applied Ecology, 35, 422–433.

[plb70197-bib-0020] Hoffmann W.A. (2000) Post‐establishment seedling success in the Brazilian Cerrado: a comparison of savanna and forest species. Biotropica, 32, 62–69.

[plb70197-bib-0021] Hoffmann W.A. , Orthen B. , Franco A.C. (2004) Constraints to seedling success of savanna and forest trees across the savanna‐forest boundary. Oecologia, 140, 252–260. 15148603 10.1007/s00442-004-1595-2

[plb70197-bib-0022] Hovenden M.J. , Newton P.C.D. , Wills K.E. , Janes J.K. , Williams A.L. , Vander Schoor J.K. , Nolan M.J. (2008) Influence of warming on soil water potential controls seedling mortality in perennial but not annual species in a temperate grassland. New Phytologist, 180, 143–152. 18631296 10.1111/j.1469-8137.2008.02563.x

[plb70197-bib-0023] Kitajima K. (1992) Relationship between photosynthesis and thickness of cotyledons for tropical tree species. Functional Ecology, 6, 582.

[plb70197-bib-0024] Kitajima K. (1994) Relative importance of photosynthetic traits and allocation patterns as correlates of seedling shade tolerance of 13 tropical trees. Oecologia, 98, 419–428. 28313920 10.1007/BF00324232

[plb70197-bib-0025] Kos M. , Poschlod P. (2008) Correlates of inter‐specific variation in germination response to water stress in a semi‐arid savannah. Basic and Applied Ecology, 9, 645–652.

[plb70197-bib-0026] Ledru M.‐P. (2002) 3. Late quaternary history and evolution of the cerrados as revealed by palynological records. In: Oliveira P. , Marquis R. (Eds), The Cerrados of Brazil. Columbia University Press, New York Chichester, West Sussex, pp 33–50.

[plb70197-bib-0027] Lenth R.V. (2024) emmeans: estimated marginal means, aka least‐squares means. The R Foundation.

[plb70197-bib-0028] Lorenzi H. (1992) Árvores brasileiras: manual de identificação e cultivo de plantas arbóreas nativas do Brasil.

[plb70197-bib-0029] Ma S. , Concilio A. , Oakley B. , North M. , Chen J. (2010) Spatial variability in microclimate in a mixed‐conifer forest before and after thinning and burning treatments. Forest Ecology and Management, 259, 904–915.

[plb70197-bib-0030] Macedo M.A. , Pinhate S.B. , Bowen E.C. , Musso C. , Miranda H.S. (2021) Constraints on tree seedling establishment after fires: passing the germination bottlenecks. Plant Biology, 24, 176–184. 34546625 10.1111/plb.13335

[plb70197-bib-0031] Marcolin E. , Marzano R. , Vitali A. , Garbarino M. , Lingua E. (2019) Post‐fire management impact on natural forest regeneration through altered microsite conditions. Forests, 10, 1014.

[plb70197-bib-0032] McLaren K.P. , McDonald M.A. (2003) The effects of moisture and shade on seed germination and seedling survival in a tropical dry forest in Jamaica. Forest Ecology and Management, 183, 61–75.

[plb70197-bib-0033] Michael B.W. , Peter B.R. (2000) Seed size, nitrogen supply, and growth rate affect tree seedling survival in deep shade. Ecological Society of America, 81, 1887–1901.

[plb70197-bib-0034] Midgley J.J. (1996) Why the World's vegetation is not totally dominated by Resprouting plants; because resprouters are shorter than reseeders. Ecography, 19, 92–95.

[plb70197-bib-0035] Milberg P. , Lamont B.B. (1997) Seed/cotyledon size and nutrient content play a major role in early performance of species on nutrient‐poor soils. New Phytologist, 137, 665–672.

[plb70197-bib-0036] Motta G.S.T. , Pilon N. , Fidelis A. , Kolb R.M. (2024) Smoke effects on the germination of Cerrado species. Plant Ecology, 225, 685–693.

[plb70197-bib-0037] Nelson C.J. , Larson K.L. (2015) Seedling growth. In: Tesar M.B. (Ed), Physiological basis of crop growth and development. American Society of Agronomy, Crop Science Society of America, Madison, WI, USA, pp 93–129.

[plb70197-bib-0038] Newberry B.M. , Power C.R. , Abreu R.C.R. , Durigan G. , Rossatto D.R. , Hoffmann W.A. (2020) Flammability thresholds or flammability gradients? Determinants of fire across savanna‐forest transitions. New Phytologist, 228, 910–921. 33410161 10.1111/nph.16742

[plb70197-bib-0039] Pausas J.G. (2017) Bark thickness and fire regime: another twist. New Phytologist, 213, 13–15. 27891644 10.1111/nph.14277

[plb70197-bib-0040] Pilon N.A.L. , Buisson E. , Durigan G. (2018) Restoring Brazilian savanna ground layer vegetation by topsoil and hay transfer. Restoration Ecology, 26, 73–81.

[plb70197-bib-0041] Ratter J.A. , Bridgewater S. , Ribeiro J.F. (2003) Analysis of the floristic composition of the brazilian cerrado vegetation III: comparison of the woody vegetation of 376 areas. Edinburgh Journal of Botany, 60, 57–109.

[plb70197-bib-0042] Ribeiro L.C. , Borghetti F. (2014) Comparative effects of desiccation, heat shock and high temperatures on seed germination of savanna and forest tree species. Austral Ecology, 39, 267–278.

[plb70197-bib-0043] Ribeiro L.C. , Pedrosa M. , Borghetti F. (2013) Heat shock effects on seed germination of five Brazilian savanna species. Plant Biology, 15, 152–157. 22672775 10.1111/j.1438-8677.2012.00604.x

[plb70197-bib-0044] Rossatto D.R. , Hoffmann W.A. , Carvalho Ramos Silva L. , Haridasan M. , Sternberg L.S.L. , Franco A.C. (2013) Seasonal variation in leaf traits between congeneric savanna and forest trees in Central Brazil: implications for forest expansion into savanna. Trees, 27, 1139–1150.

[plb70197-bib-0045] Sales N.M. , Pérez‐García F. , Silveira F.A.O. (2013) Consistent variation in seed germination across an environmental gradient in a Neotropical savanna. South African Journal of Botany, 87, 129–133.

[plb70197-bib-0046] Schupp E.W. (1995) Seed‐seedling conflicts, habitat choice, and patterns of plant recruitment. American Journal of Botany, 82, 399.

[plb70197-bib-0047] Schupp E.W. , Jordano P. , Gómez J.M. (2010) Seed dispersal effectiveness revisited: a conceptual review. New Phytologist, 188, 333–353. 20673283 10.1111/j.1469-8137.2010.03402.x

[plb70197-bib-0048] Scott K. , Setterfield S. , Douglas M. , Andersen A. (2010) Soil seed banks confer resilience to savanna grass‐layer plants during seasonal disturbance. Acta Oecologica, 36, 202–210.

[plb70197-bib-0049] Setterfield S.A. (2002) Seedling establishment in an Australian tropical savanna: effects of seed supply, soil disturbance and fire. Journal of Applied Ecology, 39, 949–959.

[plb70197-bib-0050] Silva J.F. , Castro F. (1989) Fire, growth and survivorship in a Neotropical savanna grass Andropogon semiberbis in Venezuela. Journal of Tropical Ecology, 5, 387–400.

[plb70197-bib-0052] Souza T.V. , Torres I.C. , Steiner N. , Paulilo M.T.S. (2015) Seed dormancy in tree species of the tropical Brazilian Atlantic Forest and its relationships with seed traits and environmental conditions. Revista Brasileira de Botânica, 38, 243–264.

[plb70197-bib-0053] Venables W.N. , Ripley B.D. (2002) Modern applied statistics with S, 4th edition. Springer, New York. 10.1007/978-0-387-21706-2

[plb70197-bib-0054] Wood S.N. (2003) Thin plate regression splines. Journal of the Royal Statistical Society. Series B, Statistical Methodology, 65, 95–114.

[plb70197-bib-0055] Zirondi H.L. , Silveira F.A.O. , Fidelis A. (2019) Fire effects on seed germination: heat shock and smoke on permeable vs impermeable seed coats. Flora, 253, 98–106.

[plb70197-bib-0056] Zuloaga‐Aguilar S. , Briones O. , Orozco‐Segovia A. (2011) Seed germination of montane forest species in response to ash, smoke and heat shock in Mexico. Acta Oecologica, 37, 256–262.

[plb70197-bib-0057] Zuur A. , Ieno E.N. , Walker N. , Saveliev A.A. , Smith G.M. (2009) Mixed effects models and extensions in ecology with R. Springer, New York.

