# Supplementary material for: Effects of post‐fire on seed germination and seedling recruitment of a generalist savanna woody species
Source: Plant Biol (Stuttg). 2026 Mar 5;28(4):1190–7. doi: 10.1111/plb.70197 (PMC13175954; doi:10.1111/plb.70197)
Supplement: Supplementary file 1 — Table S1. Predicted probabilities of seed germination and mortality obtained from the generalized linear model (GLM) fitted with a binomial distribution and logit link function. The model evaluated the effects of fire treatment and vegetation on germination outcomes. Predictions were generated using the predict () function with the argument type = ‘response’, which provides the estimated probability (on the response scale) of germination or death for each combination of factors based on the fitted model. [file PLB-28-1190-s002.docx]

Suplementary table 1. Predicted probabilities of seed germination and mortality obtained from the Generalized Linear Model (GLM) fitted with a binomial distribution and logit link function. The model evaluated the effects of fire treatment and vegetation on germination outcomes. Predictions were generated using the predict () function with the argument type = "response", which provides the estimated probability (on the response scale) of germination or death for each combination of factors based on the fitted model.

| Vegetation | Treatment | Probability germination | Probability death |
| --- | --- | --- | --- |
| Savanna | B | 0.08 | 0.45 |
|  | UB | 0.15 | 0.50 |
| Transition | B | 0.53 | 0.21 |
|  | UB | 0.46 | 0.22 |
| Forest | B | 0.75 | 0.21 |
|  | UB | 0.64 | 0.22 |
