# Supplementary material for: Effects of post‐fire on seed germination and seedling recruitment of a generalist savanna woody species
Source: Plant Biol (Stuttg). 2026 Mar 5;28(4):1190–7. doi: 10.1111/plb.70197 (PMC13175954; doi:10.1111/plb.70197)
Supplement: Supplementary file 2 — Table S2. Monthly median number of leaves per seedling across vegetation types and treatments (B = burned; UB = unburned) over the 7‐month study period, accounting for repeated measurements of the same individuals. N. seedlings indicate the number of seedlings observed per month (number total expected: 60). [file PLB-28-1190-s001.docx]

**Supplementary Table 2.** Monthly median number of leaves per seedling across vegetation types and treatments (B = burned; UB = unburned) over the seven-month study period, accounting for repeated measurements of the same individuals. *N.seedlings* indicate the number of seedlings observed per month (Number total expected: 60).

| **Transect** | **Treatment** | **Month** | **median_leaves** | **n_seedlings** |
| --- | --- | --- | --- | --- |
| **Savanna** | **B** | **Dec_2** | **0** | **12** |
| **Savanna** | **B** | **Jan_23** | **0** | **12** |
| **Savanna** | **B** | **Feb_23** | **0** | **14** |
| **Savanna** | **B** | **Mar_23** | **0** | **12** |
| **Savanna** | **B** | **Abr_23** | **2** | **1** |
| **Savanna** | **B** | **May_2** | **0** | **4** |
| **Savanna** | **B** | **Jun_23** | **0** | **4** |
| **Savanna** | **UB** | **Dec_2** | **0** | **12** |
| **Savanna** | **UB** | **Jan_23** | **0** | **12** |
| **Savanna** | **UB** | **Feb_23** | **0** | **12** |
| **Savanna** | **UB** | **Mar_23** | **0** | **12** |
| **Savanna** | **UB** | **Abr_23** | **NA** | **0** |
| **Savanna** | **UB** | **May_2** | **0** | **4** |
| **Savanna** | **UB** | **Jun_23** | **NA** | **0** |
| **Transition** | **B** | **Dec_23** | **0** | **12** |
| **Transition** | **B** | **Jan_23** | **0** | **12** |
| **Transition** | **B** | **Feb_23** | **0** | **16** |
| **Transition** | **B** | **Mar_23** | **0** | **17** |
| **Transition** | **B** | **Abr_23** | **1** | **15** |
| **Transition** | **B** | **May_23** | **2** | **13** |
| **Transition** | **B** | **Jun_23** | **2** | **6** |
| **Transition** | **UB** | **Dec_22** | **0** | **12** |
| **Transition** | **UB** | **Jan_23** | **0** | **12** |
| **Transition** | **UB** | **Feb_23** | **0** | **13** |
| **Transition** | **UB** | **Mar_23** | **0** | **14** |
| **Transition** | **UB** | **Abr_23** | **2** | **11** |
| **Transition** | **UB** | **May_23** | **2** | **8** |
| **Transition** | **UB** | **Jun_23** | **2** | **6** |
| **Forest** | **B** | **Dec_22** | **0** | **12** |
| **Forest** | **B** | **Jan_23** | **0** | **18** |
| **Forest** | **B** | **Feb_23** | **0** | **42** |
| **Forest** | **B** | **Mar_23** | **0** | **40** |
| **Forest** | **B** | **Abr_23** | **0** | **38** |
| **Forest** | **B** | **May_23** | **2** | **29** |
| **Forest** | **B** | **Jun_23** | **2** | **27** |
| **Forest** | **UB** | **Dec_22** | **0** | **12** |
| **Forest** | **UB** | **Jan_23** | **0** | **15** |
| **Forest** | **UB** | **Feb_23** | **0** | **24** |
| **Forest** | **UB** | **Mar_23** | **0** | **33** |
| **Forest** | **UB** | **Abr_23** | **0** | **31** |
| **Forest** | **UB** | **May_23** | 2 | 21 |
| **Forest** | **UB** | **Jun_23** | 2 | 18 |
